# From parasite chromatin to host immune reprogramming: linking developmental state, effector regulation, and persistence in *Toxoplasma gondii*

**DOI:** 10.3389/fimmu.2026.2000150

**Published:** 2026-10-15

**Authors:** Yebom Kim, Yu Seong Ham, Jihyeon Yun, Seungbin Han, Chul-Su Yang

**Affiliations:** 1 Department of Molecular and Life Science, Hanyang University, Ansan, Republic of Korea; 2 Department of Molecular Medicine, School of Biopharmaceutical Convergence, Hanyang University, Ansan, Republic of Korea; 3 Biopeptics Inc., Ansan, Republic of Korea

**Keywords:** developmental regulation, host immune reprogramming, parasite chromatin, parasite effectors, *Toxoplasma gondii*, transcriptional regulation

## Abstract

*Toxoplasma gondii* establishes persistent infection despite sustained cell-mediated immune pressure, requiring coordinated regulation of parasite development and host immune responses. Parasite developmental states are controlled by sequence-specific transcription factors and chromatin regulators, including apicomplexan AP2 proteins and ATP-dependent chromatin remodelers, which establish stage-specific transcriptional programs. These developmental programs are accompanied by changes in the expression and delivery of host-modulatory effectors, although effector abundance does not necessarily predict their delivery into host cells. This review examines the emerging mechanistic link between parasite-intrinsic transcriptional and chromatin states, stage-dependent effector regulation, and host immune reprogramming during *T. gondii* infection. We discuss how rhoptry and dense-granule effectors alter host signaling, STAT-dependent transcription, transcriptional cofactor and corepressor function, histone modification, and chromatin accessibility. These activities can suppress interferon-responsive and cell-death pathways or induce inflammatory and migration-associated transcriptional programs, with outcomes that depend on the combination of effectors, host cell identity, and parasite genetic background. We further consider how stage-specific effector activity contributes to immune containment, persistence, reactivation, and tissue pathology in the central nervous system, ocular tissues, and placenta. Although individual parasite transcriptional regulators and host-targeting effectors have been characterized in considerable detail, direct regulatory connections between parasite developmental chromatin states and stage-specific effector expression and delivery remain incompletely defined. Defining these connections across parasite stages, host cell types, genetic backgrounds, and infected tissues will be important for understanding how parasite developmental programs shape tissue-specific immune modulation and pathogenesis.

## Highlights

- Parasite chromatin programs establish developmental states that shape host-facing gene expression in *Toxoplasma gondii*.
- Developmental transitions alter the expression and delivery of parasite effectors, linking parasite state to host immune modulation.
- *T. gondii* effectors reprogram host immune transcription through STAT signaling, transcriptional cofactors, corepressors, and chromatin regulation.
- Integrated effector activities generate context-dependent immune outcomes that vary across host cells, parasite strains, and infected tissues.
- Defining the links between parasite developmental regulation and effector activity is critical for understanding persistence, reactivation, and tissue pathology.

## 1 Introduction

*Toxoplasma gondii* establishes long-term infection in mammalian hosts despite sustained cell-mediated immune pressure (1, 2). Rapidly replicating tachyzoites drive parasite expansion and dissemination, whereas bradyzoites persist within tissue cysts during chronic infection (3–5) (**Box 1**). Persistent infection is particularly consequential in the central nervous system (CNS), ocular tissues, and the placenta, which are major sites of clinically important toxoplasmosis (6, 7). Tachyzoites and bradyzoites share the same genome but exhibit markedly different transcriptional profiles (3, 8). Host immunity encounters parasites whose host-facing phenotype varies with developmental state (9–11) as well as genetic background (12–14).

Box 1 **Developmental transitions across the *Toxoplasma gondii* life cycle.**

*  Toxoplasma gondii* alternates among rapidly replicating tachyzoites, persistent bradyzoites, and enteric sexual stages that support dissemination, chronic infection, and transmission (85). Following transmission to an intermediate host, sporozoites or bradyzoites convert to tachyzoites, which undergo repeated cycles of host cell invasion, intracellular replication, egress, and reinvasion (85). A subset of tachyzoites subsequently differentiates into bradyzoites and establishes tissue cysts (85). Tachyzoites and bradyzoites are transcriptionally distinct, and stage conversion is accompanied by broad reorganization of parasite gene expression (3, 85).

Bradyzoite differentiation is regulated by a bradyzoite formation deficient 1 (BFD1)–bradyzoite formation deficient 2 (BFD2) positive-feedback circuit (3, 47). BFD1 is necessary for differentiation and sufficient to induce a broad bradyzoite transcriptional program, while BFD2 promotes BFD1 translation and reinforces the chronic-stage program (3, 47). Bradyzoites within tissue cysts constitute the long-lived stage of chronic infection and resume tachyzoite replication during reactivation (85). Host cell export of selected effectors is retained during bradyzoite differentiation. MYR1 was detected in developing cysts, and *Toxoplasma* inhibitor of STAT1-dependent transcription (TgIST) continued to reach the host nucleus during prolonged bradyzoite differentiation and attenuated interferon-γ-induced interferon regulatory factor 1 expression. Host-nuclear accumulation of the other examined effectors declined with distinct kinetics (10).

Sexual development normally occurs in the feline intestinal epithelium (85). After ingestion of tissue cysts, bradyzoites invade intestinal epithelial cells and undergo merogony, followed by gametogenesis and formation of unsporulated oocysts (85). In tachyzoites, TgAP2XI-2 and TgAP2XII-1 repress merozoite-associated genes together with *T. gondii* microrchidia (TgMORC) and histone deacetylase 3 (TgHDAC3) (26). Simultaneous depletion of the two apicomplexan AP2 (ApiAP2) factors induces extensive transcriptional reprogramming toward a merozoite-like pre-sexual state (26). Host intestinal lipid metabolism contributes to the restricted host range of sexual development, as inhibition of delta-6-desaturase combined with dietary linoleic acid permits sexual development and oocyst shedding in mice (86). After shedding, oocysts sporulate in the environment and become infectious (85). Ingested sporozoites then initiate infection in intermediate hosts (85).

Within the parasite nucleus, developmental identity is maintained by sequence-specific transcription factors and chromatin regulators. Apicomplexan AP2 (ApiAP2) factors and ATP-dependent chromatin remodelers coordinate stage- and cell-cycle-associated transcription (15–21), whereas repressive ApiAP2 factors and chromatin regulators restrict competing developmental programs (17, 22–24). Promoter accessibility is not equivalent to transcriptional activation (18, 20, 21), and expression of alternative-stage genes can occur without stable developmental commitment (24, 25). Developmental reprogramming extends to host-interacting genes, linking parasite developmental state to host-facing gene expression (9, 20, 26).

Effector production alone does not define host-modulatory activity; parasite proteins must reach the cellular compartment in which they act. Rhoptry (ROP) effectors are injected into host cells during invasion (27), whereas selected dense-granule (GRA) effectors are secreted into the parasitophorous vacuole after invasion and subsequently translocated across the vacuole membrane to reach the host cytosol or nucleus (28, 29). At cytokine-responsive loci, transcriptional output depends on transcription-factor activity, cofactor recruitment, and the local chromatin environment (1, 30–32). *T. gondii* effectors alter signal transducer and activator of transcription (STAT)-dependent transcription, host corepressor function, histone modification, and chromatin accessibility (30, 31, 33–35). Their effects are not uniformly suppressive but instead range from attenuation of interferon (IFN)-responsive and cell-death programs to induction of inflammatory and migration-associated transcriptional programs that facilitate parasite dissemination (27, 33, 35, 36). The resulting host transcriptional response reflects the combined activity of multiple parasite effectors as well as the host cell context in which they operate (12, 37).

At selected secretory loci, parasite chromatin regulation is linked to promoter accessibility and transcript abundance (20). Effector expression and delivery to host cells change during parasite differentiation (9–11, 38), and distinct parasite developmental states are accompanied by different host transcriptional responses (11, 39). During bradyzoite development, Myc regulation 1 (MYR1)-dependent export is retained (10, 11) and contributes substantially to host transcriptional remodeling, with *Toxoplasma* inhibitor of STAT1-dependent transcription (TgIST) accounting for part of the suppression of interferon gamma (IFN-γ)-responsive transcription (11). It remains unclear which effector loci are directly controlled by parasite chromatin and transcriptional regulators during development and how stage-specific patterns of effector expression and delivery shape host immune transcription and chromatin regulation across host cell types and tissues. This review examines the parasite transcriptional and chromatin programs that establish developmental states, their relationship to host-modulatory effector expression and delivery, and the mechanisms by which delivered effectors reshape host immune transcription and chromatin regulation, with consequences for immune containment and tissue pathology. We focus on mammalian infection stages and consider alternative developmental states when relevant to transcriptional restraint or stage conversion.

## 2 Parasite chromatin programs organize infection-competent transcriptional states

*T. gondii* exists in developmentally distinct transcriptional states during infection (3, 9, 22). Parasite replication, invasion, and secretion depend on stage-appropriate gene expression, which is extensively reorganized during developmental transitions (5, 15, 18, 19, 26). Chromatin remodelers and sequence-specific transcription factors establish and maintain these states, which are accompanied by gene-expression programs that include host-facing secretory genes (9, 20–23). Parasite-intrinsic regulation provides the transcriptional context in which stage-associated changes in host-modulatory effector expression emerge, linking parasite developmental state to changes in its host-facing repertoire during infection (9–11, 20).

### 2.1 Promoter competence and transcriptional activation

ATP-dependent switch/sucrose non-fermentable (SWI/SNF) remodeling sustains promoter accessibility, transcription, and lytic growth in tachyzoites (20, 21). TgSNF2a and TgSNF2b were initially characterized as SWI/SNF-family ATPases occupying promoters of numerous tachyzoite-expressed genes (20). Co-immunoprecipitation in the same study detected an association between the two ATPases but could not distinguish a single complex from separate, cooperating assemblies (20). A subsequent TgSWI3-centered analysis defined two complexes containing mutually exclusive TgSNF2a or TgSNF2b ATPases together with shared core subunits (21). The two complexes also occupy different positions within promoter chromatin. TgSNF2b is preferentially associated with the −1 and +1 nucleosomes flanking the nucleosome-depleted region, whereas TgSNF2a is shifted downstream toward the +1 and +2 nucleosomes relative to the transcription start site (21) (**Figure 1B**). Across the cell cycle, phase-regulated promoters show a shift from TgSNF2b toward TgSNF2a binding as they move from a poised to an active state (21). The transition supports a broader role for TgSNF2b in maintaining transcriptional competence, whereas TgSNF2a is more closely associated with phase-specific transcriptional activation (21). TgSNF2b also colocalizes with *T. gondii* microrchidia (TgMORC) at a subset of developmentally regulated promoters, consistent with its presence at transcriptionally poised or repressed loci as well as transcriptionally active genes (20, 21). Promoter accessibility and productive transcription are separable features of tachyzoite promoter regulation (20, 21).

**Figure 1:**
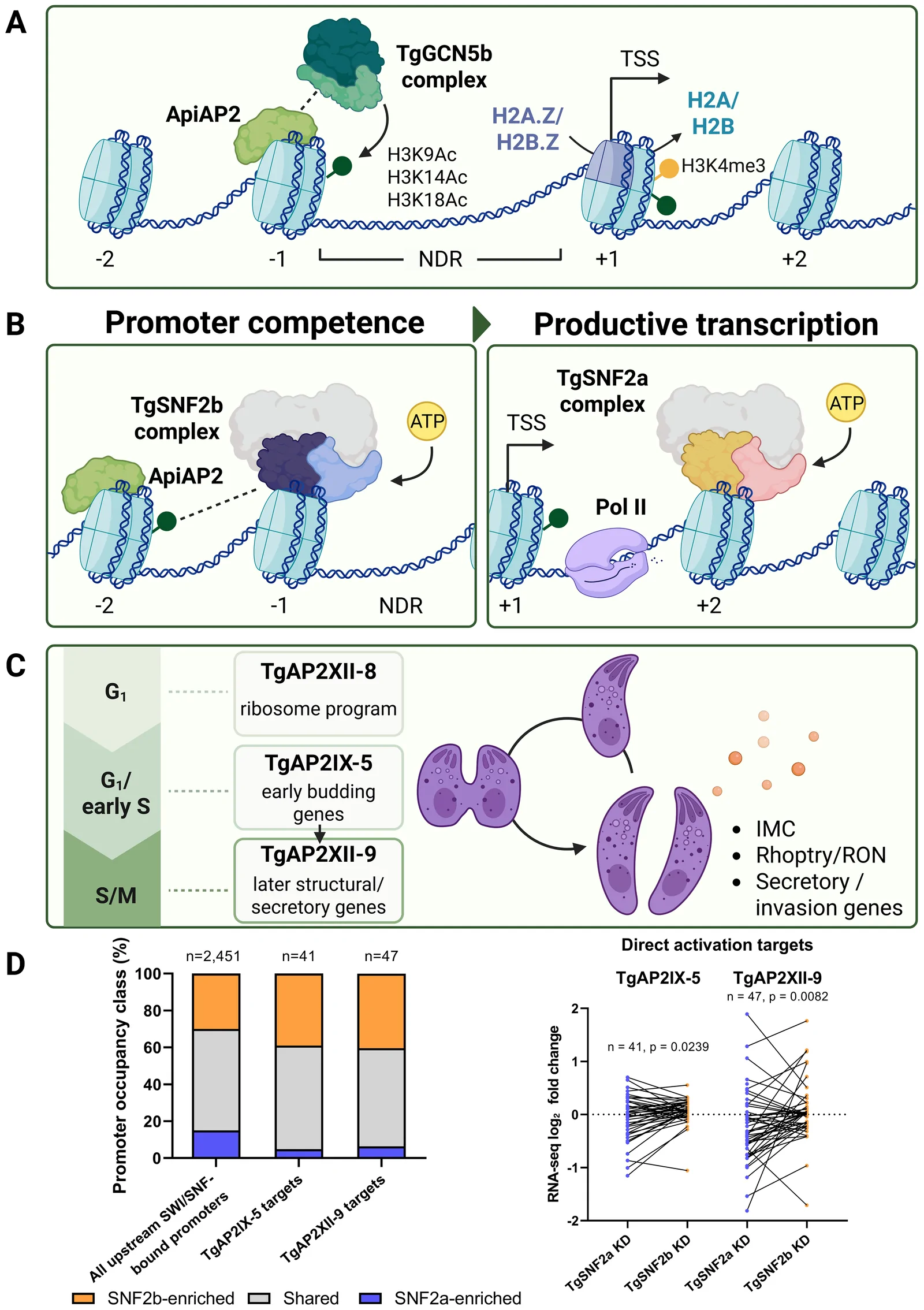
Chromatin remodeling and ApiAP2 control of tachyzoite transcription in *Toxoplasma gondii*. **(A)** Transcriptionally active promoter chromatin is associated with TgGCN5b-mediated histone H3 acetylation, H2A.Z/H2B.Z-containing nucleosomes, and histone methylation. TgGCN5b-containing complexes interact with ApiAP2 factors to promote H3 acetylation. **(B)** Distinct SWI/SNF complexes containing TgSNF2b or TgSNF2a occupy different promoter-proximal nucleosomes. TgSNF2b occupancy at the −1 and +1 nucleosomes is associated with promoter competence, whereas TgSNF2a occupancy toward the +1 and +2 nucleosomes accompanies productive transcription and RNA polymerase II engagement; ATP indicates the catalytic ATPase activity of each complex. **(C)** Cell-cycle-regulated ApiAP2 factors provide sequence-specific control of tachyzoite transcription. TgAP2XII-8 acts around the C/G1 transition, whereas TgAP2IX-5 directly activates *TgAP2XII-9*, linking an earlier budding program to the later S/M-phase transcriptional program. TgAP2XII-9 regulates genes associated with daughter cell maturation, secretory organelles, and effector expression. Panels A–C represent intersecting regulatory layers and do not imply a defined temporal or causal hierarchy among chromatin modifications, SWI/SNF remodeling, and ApiAP2-mediated regulation. **(D)** Comparison of SWI/SNF promoter occupancy and transcriptional dependency among genes directly activated by TgAP2IX-5 or TgAP2XII-9. Top, stacked bars show the proportions of upstream SWI/SNF-bound promoters classified as TgSNF2a-enriched, shared, or TgSNF2b-enriched. Both target sets were predominantly associated with shared or TgSNF2b-enriched promoters, although their occupancy-class distributions did not differ significantly from that of the background set (Fisher’s exact test). Bottom, paired RNA-seq log_2_ fold changes for the same target genes following 6 h of depletion of TgSNF2a or TgSNF2b. TgAP2IX-5 and TgAP2XII-9 targets showed significantly lower expression after TgSNF2a depletion than after TgSNF2b depletion (*p* = 0.0239 and *p* = 0.0082, respectively; two-tailed Wilcoxon matched-pairs signed-rank test), indicating that promoter occupancy class and early transcriptional dependency do not necessarily coincide. Numbers above the plots indicate the number of genes included in each analysis. Panel **(D)** was generated by reanalysis of published datasets (15, 19, 21). ApiAP2, apicomplexan AP2; pol II, RNA polymerase II; C, cytokinesis; G1, first gap phase; S, DNA synthesis phase; M, mitosis; NDR, nucleosome-depleted region; RNA-seq, RNA sequencing; SWI/SNF, switch/sucrose non-fermentable; TSS, transcription start site. Image created with BioRender.com.

TgGCN5b is a lysine acetyltransferase required for tachyzoite proliferation (40). Expression of a catalytically inactive TgGCN5b variant reduces histone H3 acetylation at lysines 9 and 14 (H3K9ac and H3K14ac) while lowering expression of TgGCN5b-associated genes (40). Proteomic analysis of TgGCN5b-associated proteins also recovered two putative SWI/SNF ATPases that were subsequently characterized as TgSNF2a and TgSNF2b (20, 21, 40). Their recovery in TgGCN5b-associated proteomes links the acetyltransferase machinery biochemically to SWI/SNF components without establishing functional or directional dependence between them (20, 21, 40).

Early epigenomic mapping identified histone H3 lysine 4 trimethylation (H3K4me3) and histone acetylation as characteristic features of active *T. gondii* promoters (41), and histone H4 lysine 31 acetylation (H4K31ac) was subsequently linked to transcriptionally active promoter chromatin (42) (**Figure 1A**). TgSNF2a shows positive associations with H3K4me3, H3K14ac, and H4K31ac, whereas TgSNF2b is associated with a broader range of chromatin states, including poised or repressed loci (21). H2A.Z/H2B.Z-containing nucleosomes are enriched around active promoters, where they colocalize with H3K4me3 (43). *T. gondii* bromodomain protein 4 (TgBDP4) was enriched in TgSNF2a- and TgSNF2b-associated proteomes (20) and was independently shown to bind acetylated histone H3 (44). Its association with SWI/SNF components and recognition of acetylated histones make TgBDP4 a candidate acetyl-lysine reader at SWI/SNF-associated chromatin, although direct recruitment of either complex through TgBDP4 has not been demonstrated (20, 44). The available data connect histone acetylation and SWI/SNF remodeling at the levels of protein association and chromatin context, but do not define a linear TgGCN5b–TgBDP4–SWI/SNF recruitment pathway (20, 21, 40, 44).

Promoter competence denotes a permissive state rather than active transcription itself. At poised developmental promoters, TgSNF2b remains associated with chromatin while transcription is temporally or developmentally restrained (20, 21). The transition toward active transcription is accompanied by reduced TgMORC occupancy, increased H3K14ac, and greater TgSNF2a association (21). The upstream signals that initiate such promoter-state transitions remain unresolved. Sequence-specific transcription factors help specify which competent promoters become transcriptionally active (15, 18, 19).

### 2.2 ApiAP2 regulatory cascades coordinate tachyzoite transcription

Promoter competence alone does not determine which genes proceed to active transcription. Sequence-specific ApiAP2 factors add a further level of selectivity by engaging distinct promoter sets during tachyzoite proliferation (15, 18, 19). TgAP2XII-8 is expressed around the cytokinesis-to-G1 (C/G1) transition and binds broadly to promoter-proximal chromatin, with 97% of its assigned target genes overlapping accessible regions (18). Yet only 120 of 343 high-confidence bound genes showed altered expression after acute TgAP2XII-8 depletion (18). Among the genes activated directly by TgAP2XII-8, 44 encode ribosomal proteins and form a coordinated early-G1 regulon enriched for a TgAP2XII-8-bound promoter motif (18). TgAP2XII-8 depletion causes G1-stage accumulation and prevents continued parasite proliferation (18). The contrast between broad promoter binding and a more restricted transcriptional response indicates that neither promoter accessibility nor ApiAP2 binding alone is sufficient to drive transcriptional activation (18, 21).

ApiAP2 regulation proceeds through temporally ordered transcription-factor cascades (15, 19). TgAP2IX-5 initiates daughter budding and directly activates several ApiAP2 genes, including *TgAP2XII-9* (19) (**Figure 1C**). TgAP2IX-5 depletion reduces TgAP2XII-9 expression, placing TgAP2XII-9 downstream in the cell-cycle transcriptional hierarchy (19). TgAP2XII-9 is expressed later, during DNA synthesis and mitosis (S/M), and regulates genes involved in subsequent daughter cell construction and secretory-organelle formation (15). Its direct regulon is largely distinct from that of TgAP2IX-5: only 18 of 300 TgAP2XII-9 direct targets were also targeted by TgAP2IX-5 (15). The cascade thus transfers control from an early budding program to a later transcriptional program rather than extending the same promoter repertoire (15, 19).

The precise contribution of TgAP2XII-9 to individual secretory-gene classes differs between studies. Acute-depletion analysis identified TgAP2XII-9 as a repressor of subsets of ROP, rhoptry neck (RON), and inner membrane complex (IMC) genes while activating microneme (MIC) and dense-granule GRA genes (15). A separate study linked TgAP2XII-9 binding to *RON2*, *RON4*, *RON8*, and IMC loci and observed reduced expression of ROP, RON, and IMC genes after TgAP2XII-9 loss (45). The two studies profiled transcription after 2 and 6 hours of auxin-induced depletion and after 24 hours of depletion, respectively. Because daughter-cell assembly defects were already evident after 6 hours of TgAP2XII-9 depletion, the different depletion intervals complicate direct comparison of the transcriptional responses (15, 45). Despite these differences, both studies place TgAP2XII-9 in the S/M-phase program that coordinates daughter cell maturation, secretory-organelle formation, and invasion-related functions (15, 45).

The relationship between ApiAP2 cascades and chromatin remodeling remains less well defined. Biochemical analyses identified multiple ApiAP2 factors within TgGCN5b-containing transcriptional complexes (46). Reanalysis of published genomic datasets showed that direct activation targets of TgAP2IX-5 and TgAP2XII-9 were enriched among SWI/SNF-bound promoters relative to the genome-wide background (15, 19–21) (**Figure 1D**). Within the SWI/SNF-bound subset, neither target set was preferentially distributed among TgSNF2a-enriched, shared, or TgSNF2b-enriched promoter classes (15, 19, 21). Expression changes were also more negative after TgSNF2a depletion than after TgSNF2b depletion, with a larger median difference among TgAP2XII-9 targets (15, 19, 21). Promoter class, however, did not map directly onto the relative transcriptional response to depletion of the two ATPases. The cross-dataset patterns do not establish whether ApiAP2 binding directs recruitment of the TgSNF2a- or TgSNF2b-containing SWI/SNF complex (15, 19–21).

Combined depletion of TgSNF2a and TgSNF2b reduces promoter accessibility at multiple ApiAP2 genes (20). Expression changes following SWI/SNF depletion may arise from both local effects at target promoters and indirect changes mediated through ApiAP2-dependent transcription (20, 21). The available data support functional interplay between SWI/SNF remodeling and ApiAP2-dependent transcription, but do not yet resolve how either system directs the other at individual promoters (15, 19–21). Maintaining the tachyzoite state requires continued repression of competing developmental programs.

### 2.3 Developmental restraint and commitment

Developmental restraint in tachyzoites involves promoter-proximal chromatin remodeling together with stage-specific transcriptional repression (16, 17, 22). The imitation switch family remodelers TgSNF2h and TgSNF2L contribute to this regulation through distinct effects on promoter chromatin (16, 17). TgSNF2h maintains promoter-proximal chromatin organization, and its depletion reduces TgMORC binding at protein-coding promoters (16). Depletion of TgSNF2L derepresses genes characteristic of enteroepithelial stages, merozoites, and gametes and induces merogony-like division patterns (17). Genes derepressed after TgSNF2L depletion overlap substantially with developmental genes regulated by the TgMORC–TgHDAC3 (histone deacetylase 3) repressive system (17, 22). Stage-regulatory ApiAP2 factors contribute promoter specificity to this repression (23, 26). TgAP2XI-2 and TgAP2XII-1 bind promoters of merozoite-specific genes and recruit TgMORC and TgHDAC3 to maintain repression at these loci (23, 26). Combined depletion of the two ApiAP2 factors reduces TgMORC and TgHDAC3 enrichment at target promoters while increasing local chromatin accessibility and transcription of merozoite and pre-gamete genes (26).

Weakening developmental restraint, however, does not necessarily produce stable commitment to an alternative fate. Loss of TgAP2IV-4 permits premature expression of bradyzoite surface-antigen and cyst-wall genes while parasites remain replicative (24). TgAP2IV-4 binds a poly(dC):poly(dG)-rich region of the *SRS9* promoter, directly linking its sequence-specific DNA binding to repression of a bradyzoite locus during tachyzoite growth (24). *In vivo*, TgAP2IV-4-deficient parasites recruit increased numbers of Ly6C^hi^ inflammatory monocytes and fail to establish tissue cysts in the brain (24). Differentiation-permissive Tg68 tachyzoites constitutively express key bradyzoite regulators, including bradyzoite formation deficient 1 (BFD1) and bradyzoite formation deficient 2 (BFD2), together with multiple bradyzoite-specific genes (25). After stress-induced differentiation, Tg68 acquires transcriptional features of mature bradyzoites at early time points (25). In both settings, expression of bradyzoite-associated genes precedes or occurs without stable commitment to the bradyzoite state (24, 25).

Stable commitment to bradyzoite differentiation requires positive regulatory reinforcement as well as release from tachyzoite-stage repression (3, 47). Under differentiation-inducing conditions, BFD1 is necessary for bradyzoite differentiation and is sufficient to induce a broad chronic-stage transcriptional response when ectopically expressed (3). BFD1 induces BFD2 expression, whereas BFD2 promotes BFD1 translation, forming a positive-feedback circuit that increases BFD1 accumulation during differentiation (47). The circuit stabilizes commitment to the bradyzoite state (47). Bradyzoites form tissue cysts that support long-term persistence in host tissues (4). Bradyzoite commitment marks a major reorganization of parasite developmental identity and the transcriptional context in which host-interacting genes are expressed (3, 9, 47).

## 3 From parasite transcriptional state to host-modulatory effector output

Developmental reorganization extends to the parasite secretory repertoire. Perturbation of chromatin and transcriptional regulators alters the expression of subsets of ROP and GRA genes alongside broader developmental gene sets (20, 26) (see **Table 1**). Transcriptionally distinct parasite states *in vivo* show different secretory-gene profiles (9). Host-modulatory effector expression varies selectively across parasite transcriptional states (9, 20, 26).

**Table 1:** Experimental evidence linking parasite regulators to host-modulatory effector genes in *Toxoplasma gondii*.

Parasite regulator	Host-modulatory effector(s)	Regulatory evidence	Ref.
TgSNF2a	ROP5; ROP17; ROP18	CUT&Tag promoter occupancy; expression response by depletion RNA-seq; accessibility loss by dual-depletion ATAC-seq; +1/+2 nucleosome enrichment	(20, 21)
TgSNF2b	ROP5; ROP17; ROP18	CUT&Tag promoter occupancy; expression response by depletion RNA-seq; accessibility loss by dual-depletion ATAC-seq; −1/+1 nucleosome enrichment	(20, 21)
TgAP2XII-9	ROP17	CUT&Tag promoter occupancy; inducible depletion; *ROP17* downregulation by RNA-seq	(15, 45)
TgAP2XI-2/TgAP2XII-1	ROP5; ROP16; ROP18; GRA16; GRA24; TgIST	Co-depletion; effector downregulation by RNA-seq; locus-specific promoter binding not demonstrated	(23, 26)
TgMORC	ROP16	TgMORC depletion; *ROP16* downregulation by RNA-seq; MORC/HDAC3 promoter occupancy not demonstrated	(22)
TgAP2XI-5	ROP18	ChIP-on-chip promoter occupancy; EMSA-confirmed GCTAGC motif binding; promoter motif mutagenesis; luciferase reporter assay	(84)
TgAP2XI-5	ROP5	ChIP-on-chip promoter occupancy; expression response not demonstrated	(84)
BFD1	ROP5; ROP16; ROP18; GRA15	Conditional BFD1 stabilization; transcript downregulation by RNA-seq; no assigned BFD1 CUT&RUN peak at these loci	(3)

RNA-seq, RNA sequencing; ChIP, chromatin immunoprecipitation; CUT&Tag, cleavage under targets and tagmentation; CUT&RUN, cleavage under targets and release using nuclease; ATAC-seq, assay for transposase-accessible chromatin with sequencing; EMSA, electrophoretic mobility shift assay.

Promoter-level analyses link SWI/SNF binding to chromatin accessibility and transcription at selected secretory genes. TgSNF2a and TgSNF2b co-bind 1,186 promoters, including numerous GRA and ROP loci, although only 239 of the co-bound genes show altered expression after depletion (20). Combined depletion reduces chromatin accessibility at promoters of GRA, ROP, and MIC genes (20). At the *GRA6* and *ROP18* loci, SWI/SNF-bound promoters show reduced accessibility together with lower transcript abundance after depletion (20). Established immune-modulatory effectors respond differently to SWI/SNF depletion, with no uniform dependence across the repertoire (20).

Co-depletion of TgAP2XI-2 and TgAP2XII-1 is accompanied by transcriptional changes characteristic of merozoite and pre-gamete programs (23, 26). Approximately 80% of tachyzoite-specific rhoptry proteins, including ROP5, ROP16, and ROP18, are reduced in abundance (26). GRA16, GRA24, and TgIST decline in abundance, whereas GRA11b and GRA80–82, markers of the merozoite/pre-gamete program, increase (26). The reciprocal loss of tachyzoite-associated proteins and induction of merozoite/pre-gamete GRAs parallel the extensive remodeling of secretory-gene expression observed in naturally derived merozoites (26, 48). Direct regulation of the affected tachyzoite effector loci by TgAP2XI-2 or TgAP2XII-1 has not been established (23, 26).

Single-cell RNA sequencing of bradyzoites isolated from mouse brain cysts resolved five transcriptomic groups (A–E) by unsupervised clustering (9). Group E was enriched for S/M-phase and growth-related transcripts, whereas Group A showed marked enrichment of selected rhoptry and RON transcripts without comparable S/M enrichment (9). *TgAP2XII-9* expression was elevated in both groups, and Group E showed a partially overlapping rhoptry/RON expression profile (9). TgAP2XII-9 regulates subsets of secretory genes in tachyzoites (15, 45). Direct regulation by TgAP2XII-9 has not been demonstrated in bradyzoites. Nevertheless, transcriptionally distinct bradyzoite populations identified *in vivo* differ in secretory-gene expression (9).

In recently obtained field isolates, serial *in vitro* passage altered both cystogenic phenotypes and the abundance of selected host-modulatory proteins (49, 50). Short-term culture adaptation reduced spontaneous cyst formation in several isolates (50). Proteomic analysis of the same isolate panel showed that bradyzoite and cyst markers, including BAG1, ENO1, and SRS35A, were less abundant at high passage (49). By contrast, host-modulatory effector abundance changed in an isolate-dependent manner (49). GRA15, GRA16, *Toxoplasma* E2F4-associated EZH2-inducing gene regulator (TEEGR), and *Toxoplasma* NCoR/SMRT modulator (TgNSM) increased in abundance at high passage in particular isolates, whereas the abundance of several other GRA and ROP proteins did not differ significantly between low and high passage cultures (49). Effector abundance did not change uniformly as cystogenic capacity and bradyzoite markers declined during culture adaptation (49, 50).

Stage-dependent differences are also evident in the host-cell accumulation of exported effectors. During *in vitro* differentiation, host-nuclear accumulation of GRA16, GRA24, GRA28, and TgIST declined at different rates (10). Host-nuclear GRA16 and TgIST signals remained significantly above baseline through day 6, whereas GRA24 and GRA28 were no longer elevated after days 2 and 4, respectively, despite continued detection within differentiating vacuoles (10). When fibroblasts were infected with bradyzoites harvested after six days of *in vitro* differentiation, GRA16 and TgIST were detected in host nuclei, whereas GRA24 and GRA28 were not detectably expressed (10). This reinvasion experiment assessed effector export after differentiated parasites had entered a new host cell and therefore did not test translocation across an established cyst wall. An earlier study used a tetracycline-inducible system to examine effector translocation after cyst wall formation. Expression of GRA16 and GRA24 was induced five days after the initiation of differentiation, and both proteins accumulated beneath the cyst wall without detectable host-nuclear localization (51). MYR1, required for translocation of host-targeted dense-granule effectors in tachyzoites (29), was detected in differentiating cysts throughout the *in vitro* bradyzoite time course (10). Host-nuclear TgIST accumulation, together with sustained MYR1 detection, argues against a uniform shutdown of effector export during differentiation (10). In bradyzoite-containing cells, deletion of *MYR1* markedly reduces parasite-induced changes in host transcription, while TgIST accounts for a substantial component of IFN-γ-responsive gene repression (11). Developmental progression is accompanied by changes in both effector expression and the subset of effectors that reach the host cell (10, 11). For most host-modulatory effectors, direct regulation by stage-defining transcriptional or chromatin factors has not been established (20, 26). As parasite development proceeds, host cells encounter different combinations of parasite effectors, reflecting changes in both effector abundance and delivery (9–11, 20, 26).

## 4 *Toxoplasma* effectors reshape host immune transcription by engaging transcriptional and chromatin machinery

Delivered *T. gondii* effectors target several layers of host transcriptional control, from signaling-responsive transcription factors to transcriptional cofactors, corepressor complexes, and chromatin regulators (13, 14, 30, 31, 33–35, 52–55). ROP16, GRA15, and GRA24 activate STAT3/STAT6, nuclear factor κB (NF-κB), and p38 mitogen-activated protein kinase signaling, respectively (13, 14, 53). TgIST, TgNSM, TEEGR (also known as host cyclin E 1 (HCE1)), and GRA28 engage host transcriptional cofactors, corepressor complexes, or chromatin-regulatory proteins (30, 31, 33, 34, 52, 54, 55). The combined effects on host transcription are not uniformly suppressive (12, 33, 35, 55). TgIST and TgNSM inhibit IFN-responsive or cell-death programs, whereas GRA15, GRA24, and GRA28 promote selected inflammatory or migratory responses (13, 30, 31, 33, 35, 53, 55) (**Figure 2**).

**Figure 2:**
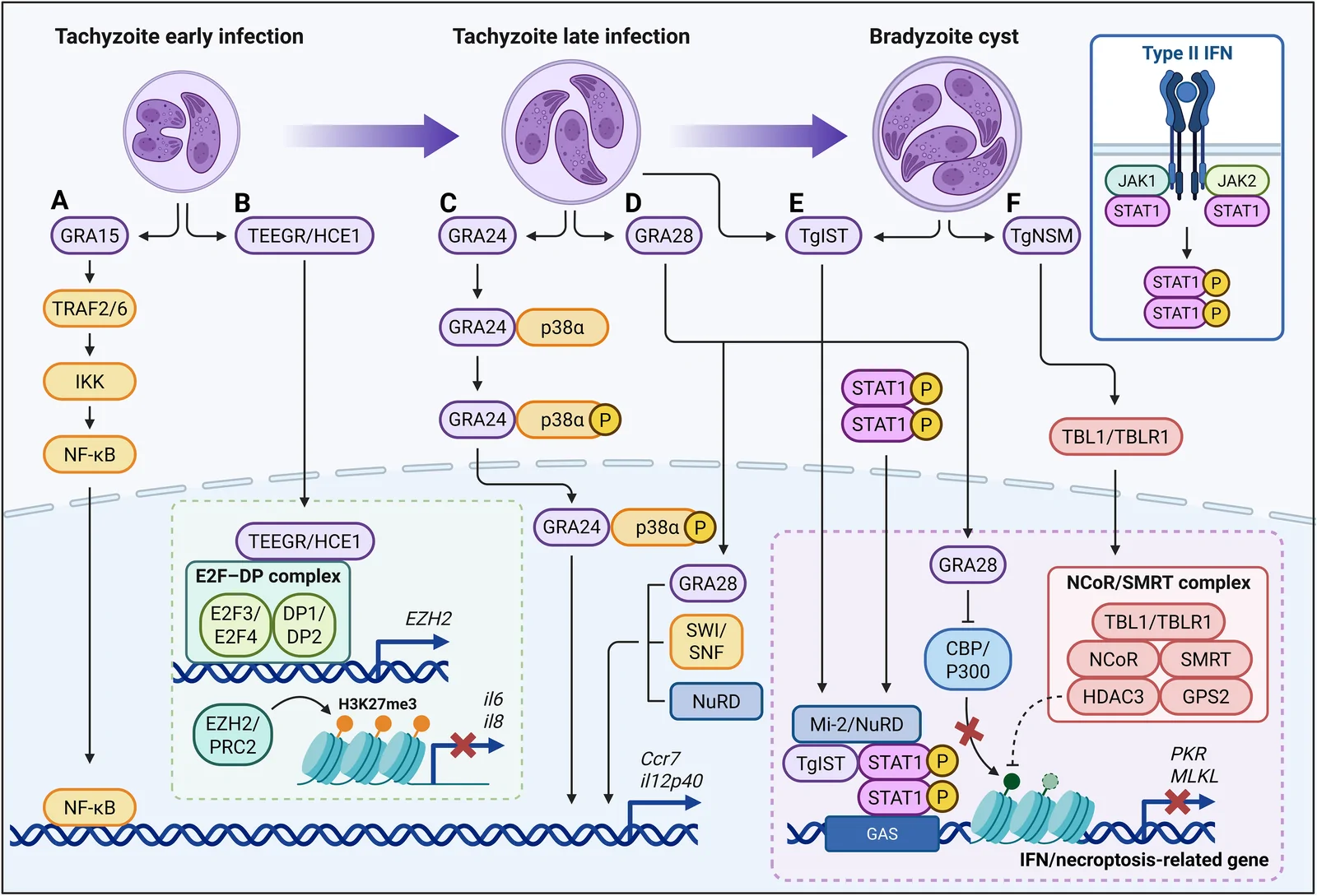
Stage-associated effector-mediated reprogramming of host immune transcription by *Toxoplasma gondii*. The upper schematic depicts parasite effectors during early tachyzoite infection, late tachyzoite infection, and the bradyzoite cyst stage. **(A)** GRA15 activates TRAF2/6–IKK–NF-κB signaling and promotes NF-κB-dependent transcription. **(B)** TEEGR/HCE1 engages the E2F–DP complex and increases *EZH2* transcription, leading to PRC2-dependent H3K27me3 deposition and repression of *IL6* and *CXCL8*. **(C)** GRA24 interacts with p38α and promotes nuclear accumulation of phosphorylated p38α, contributing to transcription of *Ccr7* and *Il12b*. **(D)** GRA28 also promotes *Ccr7* and *Il12b* transcription and interacts with host SWI/SNF and NuRD complexes. The GRA28-associated NuRD complex may differ in composition and regulatory context from the Mi-2/NuRD complex recruited by TgIST. **(E)** TgIST binds phosphorylated STAT1 dimers and recruits Mi-2/NuRD to STAT1-responsive loci. IFN-induced transcription remains repressed despite upstream STAT1 activation. The contribution of NuRD-associated chromatin regulation to this repression remains incompletely defined. The type II IFN inset depicts upstream JAK1/JAK2-dependent STAT1 phosphorylation that generates the phosphorylated STAT1 dimers targeted by TgIST. GRA28 additionally interferes with CBP/p300-dependent acetylation, providing a separate route to suppression of IFN-associated transcription. **(F)** TgNSM enhances NCoR/SMRT-mediated repression through TBL1/TBLR1, suppressing PKR and MLKL expression and related transcriptional responses. HDAC3-associated deacetylation may contribute to this repression. The chromatin-regulatory effects of TgIST, GRA28, and TgNSM converge on suppression of IFN-associated transcription but do not necessarily involve modification of the same chromatin sites or acetylated residues. CBP, CREB-binding protein; DP, dimerization partner; EZH2, enhancer of zeste homolog 2; HDAC3, histone deacetylase 3; H3K27me3, histone H3 lysine 27 trimethylation; HCE1, host cyclin E 1; IFN, interferon; IKK, IκB kinase; JAK, Janus kinase; MLKL, mixed lineage kinase domain-like protein; NCoR, nuclear receptor corepressor; NF-κB, nuclear factor κB; NuRD, nucleosome remodeling and deacetylase; PKR, protein kinase R; PRC2, Polycomb repressive complex 2; SMRT, silencing mediator for retinoid and thyroid hormone receptors; STAT1, signal transducer and activator of transcription 1; SWI/SNF, switch/sucrose non-fermentable; TBL1, transducin β-like 1; TBLR1, transducin β-like 1-related protein 1; TEEGR, *Toxoplasma* E2F4-associated EZH2-inducing gene regulator; TgIST, *Toxoplasma* inhibitor of STAT1-dependent transcription; TgNSM, *Toxoplasma* NCoR/SMRT modulator; TRAF, TNF receptor-associated factor. Image created with BioRender.com.

### 4.1 TgIST: disabling STAT1-dependent transcription

STAT1-dependent transcription is central to IFN-γ-mediated control of *T. gondii* (56). IFN-γ receptor signaling promotes STAT1 phosphorylation, homodimerization, nuclear translocation, and binding to gamma-activated sequence (GAS) elements, where STAT1 engages transcriptional coactivators including CREB-binding protein (CBP) and p300 (32). TgIST acts at a transcriptional step downstream of STAT1 activation, with STAT1 phosphorylation and nuclear accumulation preserved (30, 31). Activated STAT1 was detected at GAS-containing promoters in infected cells despite strong repression of IFN-γ-responsive transcription (30, 31).

Structural and mutational analyses localized binding of the TgIST repeat region to a surface created at the dimerized STAT1 Src homology 2 interface (57). The internal repeat region of TgIST folds into an amphipathic α helix upon association with the STAT1 dimer (57). Substitution of R380 or T390 markedly reduced STAT1 binding, and additional hydrophobic, polar, and charged residues contributed to the interaction (57). TgIST–STAT1 association reduced CBP/p300 co-precipitation with STAT1, linking this interaction to impaired coactivator recruitment (57). The repeat-containing region was sufficient to inhibit IFN-γ-induced STAT1-dependent transcription (57).

A distinct C-terminal region of TgIST associates with the Mi-2/nucleosome remodeling and deacetylase (NuRD) complex independently of the STAT1-binding region and IFN-γ stimulation (57). Current interaction data support an association between TgIST and the NuRD complex but do not identify which NuRD subunit mediates this association. Evidence for the contribution of NuRD to TgIST-mediated repression differs across experimental settings. Olias et al. detected TgIST-dependent reductions in histone H3 acetylation at GAS-containing regions of *HLA-E* and *GBP1*, indicating local changes in promoter-associated chromatin (31). Gay et al., however, found no comparable changes in H3K9ac, histone H3 lysine 27 acetylation (H3K27ac), or H4 acetylation at the STAT1-regulated loci examined, including IRF1, CXCL10, and CIITA, and inhibition of class I/II histone deacetylase activity did not relieve TgIST-dependent repression of IRF1 (30). The divergent results leave open whether NuRD-associated deacetylation is restricted to particular STAT1-responsive loci or chromatin contexts, with its broader contribution to the IFN-γ response remaining unclear (30, 31). Domain-mapping experiments further showed that a TgIST variant lacking the C-terminal NuRD-binding region retained the ability to suppress IFN-γ-induced IRF1 and major histocompatibility complex class I expression (57). Direct interference with STAT1–coactivator engagement is supported as a major repressive mechanism, whereas the contribution of NuRD-associated deacetylation or chromatin remodeling across STAT1-responsive loci remains incompletely defined (30, 31, 57).

TgIST extends its activity to STAT1/STAT2-dependent type I IFN signaling. Following IFN-β stimulation, TgIST associates with phosphorylated STAT1 and STAT2 together with NuRD components and broadly reduces IFN-responsive transcription (58). TgIST-deficient type II parasites are more susceptible to IFN-β-dependent growth restriction in human and murine macrophages, and their attenuated virulence is restored in mice lacking the type I IFN receptor (58). In sum, TgIST suppresses both type I and type II IFN responses through interactions with distinct STAT-containing complexes (30, 31, 57, 58).

### 4.2 TgNSM: co-opting host corepressors to restrain cell death

TgNSM accumulates in the host nucleus and alters both the subcellular distribution and abundance of nuclear receptor corepressor (NCoR) and silencing mediator for retinoid and thyroid hormone receptors (SMRT) complex components (33). Proteomic analysis identified transducin β-like 1-related protein 1 (TBLR1), SMRT/NCOR2, transducin β-like 1 (TBL1), and NCOR1 as prominent candidate TgNSM interactors (33). Histone deacetylase 3 (HDAC3) and G protein pathway suppressor 2 (GPS2) were detected within the same corepressor network (33). Co-immunoprecipitation further recovered SMRT and TBL1 with TgNSM (33). Ectopic TgNSM expression increased the nuclear localization of TBL1 and TBLR1 and elevated NCoR and SMRT levels, most prominently in the nuclear fraction (33). Under IFN-γ stimulation, TgNSM attenuated the induction of a subset of interferon-responsive genes (33).

Combined loss of TgNSM and TgIST derepressed a subset of interferon-responsive genes linked to regulated cell death, including genes encoding protein kinase R (PKR) and mixed lineage kinase domain-like protein (MLKL), two factors implicated in IFN-driven necroptosis (33). Ectopic TgNSM expression reduced IFN-γ- and IFN-β-induced activity of *EIF2AK2* (PKR) and *MLKL* promoter reporters (33). Combined deletion of *TgNSM* and *TgIST* increased IFN-γ-induced host cell death and cyst rupture (33). Pharmacological inhibition of PKR, RIPK1, RIPK3, or MLKL, together with genetic loss of MLKL, reduced both outcomes, implicating the necroptotic pathway in the observed cell-death phenotype (33).

GPS2, NCoR, and SMRT co-localize at IL-4-responsive enhancers and promoters in macrophages, and depletion of these corepressors increases H3K27ac and transcription at selected loci (59). SMRT was further shown to maintain the nuclear localization and chromatin binding of NCoR, HDAC3, and GPS2 (60). Loss of SMRT altered H3K27ac at inflammatory regulatory regions together with the corresponding transcriptional programs (60). The NCoR/SMRT complex has established chromatin-regulatory activity at cytokine-responsive loci, but the TgNSM study did not test whether HDAC3 or other corepressor components are recruited to the *EIF2AK2* or *MLKL* loci (33, 59, 60). Functionally, TgIST and TgNSM converge on IFN-responsive transcription through different host regulatory systems (30, 31, 33).

### 4.3 TEEGR/HCE1: EZH2-dependent repression of inflammatory transcription

TEEGR/HCE1 (TGGT1_239010), hereafter referred to as TEEGR, is a dense granule effector that accumulates in the host nucleus and engages E2F transcriptional complexes (34, 54). The protein was independently identified under the names TEEGR and HCE1. Proteomic analyses in both studies recovered E2F3, E2F4, and DP1, with DP2 additionally detected in TEEGR-containing nuclear complexes (34, 54). The study that designated the effector HCE1 established its requirement for induction of E2F-responsive genes, including cyclin E, whereas the TEEGR study linked E2F3/4 activity to regulation of enhancer of zeste homolog 2 (EZH2) (34, 54). TEEGR-dependent E2F activity induces EZH2, the catalytic subunit of Polycomb repressive complex 2 (34). During infection, E2F3 and E2F4 binding at the *EZH2* promoter increases in a TEEGR-dependent manner, placing E2F activity upstream of EZH2 induction (34). At the *IL6* and *CXCL8* (*IL8*) promoters, TEEGR-expressing parasites increase both EZH2 enrichment and H3 lysine 27 trimethylation (H3K27me3), whereas both signals decline after *TEEGR* deletion (34). Pharmacological inhibition of EZH2 methyltransferase activity restores *IL6* and *CXCL8* expression, supporting a functional role for the E2F–EZH2 axis in TEEGR-mediated repression at these inflammatory loci (34). *In vivo*, loss of TEEGR restricts parasite expansion and reduces virulence in mice, linking the E2F–EZH2 pathway to parasite persistence (34).

In bone marrow-derived macrophages, *TEEGR* deletion increased expression of *Ccr7*, *Il12b*, and *Zbtb46* relative to wild-type infection, indicating stronger pro-migratory and inflammatory transcription (35). Likewise, in brain endothelial cells and dendritic cells, loss of TEEGR enhanced GRA15–NF-κB-dependent CCL5 induction (36). EZH2 recruitment and H3K27me3 deposition were not examined at the *Ccr7* or *Ccl5* loci in either study, so the E2F–EZH2 mechanism demonstrated at *IL6* and *CXCL8* cannot be extended to all TEEGR-responsive genes (34–36). The opposing effects of TEEGR and GRA15 place host inflammatory transcription under competing parasite-derived regulatory inputs (35, 36).

### 4.4 Beyond repression: chromatin remodeling and selective host transcription

Proteomic analysis of GRA28-containing complexes recovered components of the NuRD and SWI/SNF complexes, and chromatin immunoprecipitation followed by sequencing (ChIP-seq) mapped GRA28 to multiple regions of host chromatin, including the CCR7 locus (55). GRA28-dependent CCR7 induction enabled dendritic cell-like chemotaxis in infected macrophages and contributed to their migration to secondary organs *in vivo* (55). Direct recruitment of SWI/SNF to the *CCR7* locus, however, was not demonstrated in this study (55). Subsequent assay for transposase-accessible chromatin using sequencing (ATAC-seq) in primary macrophages showed GRA28-dependent increases in chromatin accessibility at *Ccr7*, *Il12b*, and *Ccl22* (35). Comparable increases were not detected at several other cytokine, chemokine, or chemokine-receptor genes, indicating that GRA28-dependent chromatin accessibility is restricted to a subset of host genes (35).

Proteomic analysis of GRA28-containing nuclear complexes in human foreskin fibroblasts identified CBP and p300 among the most strongly enriched host proteins, and the association with p300 was further confirmed by immunoprecipitation (52). Co-expression of GRA28 with either CBP or p300 reduced H3K27ac (52). In human foreskin fibroblasts, wild-type infection likewise reduced nuclear H3K27ac relative to *Δgra28* infection, and complementation restored this phenotype (52). GRA28 reduced IFN-γ-induced GAS reporter activity in a separate transcriptional assay (52). Both GRA28 and TgIST converge on the CBP/p300 coactivator system, but their documented effects differ: TgIST impairs CBP/p300 recruitment to STAT1, whereas GRA28 reduces CBP/p300-dependent H3K27ac (52, 57). The GRA28-dependent increase in chromatin accessibility observed in primary macrophages and the reduction in H3K27ac detected in the CBP/p300 experiments were obtained under different experimental conditions, and no direct mechanistic link between the two has been demonstrated (35, 52).

In murine bone marrow-derived macrophages, GRA28-dependent chromatin accessibility operates alongside GRA15–NF-κB and GRA24–p38 signaling (35). Type II GRA15 activates canonical NF-κB, and TRAF-binding motifs in GRA15 contribute to this activity (13, 61). GRA24 directly engages and activates p38α through tandem kinase-interaction motifs, and the first motif (KIM1), spanning residues 440–455, has been structurally resolved at the p38α docking surface (53, 62). At the *Ccr7* locus, GRA15–NF-κB and GRA24–p38 signaling converge with GRA28-dependent chromatin accessibility to promote transcription and CCR7-dependent chemotaxis (35). Pharmacological inhibition further indicated a predominant contribution of NF-κB to *Ccr7* induction, with substantially less contribution from AP-1 or PU.1 (35). The accompanying increase in chromatin accessibility depends on GRA28, adding a chromatin component to the GRA15/GRA24-driven migratory response (35).

Although GRA15–NF-κB and GRA24–p38 signaling contribute to the migratory program, both pathways support protective type 1 immune responses (13, 35, 63). Type II GRA15 promotes NF-κB-dependent IL-12 production in macrophages and contributes to early control of parasite burden in mice (13). GRA24-dependent p38 activation similarly promotes IL-12 production and supports a MyD88-independent T helper 1 (Th1) response with protective activity in mice (63). By contrast, GRA15-dependent NF-κB signaling in brain endothelial cells and dendritic cells induces CCL5, and CCL5–CCR5 signaling increases the migration and microvascular sequestration of infected phagocytes, leading to greater early parasite accumulation in the brain (36). The biological consequence of effector-driven inflammatory transcription depends on the induced gene program and infected cell behavior rather than on inflammatory activation alone (13, 35, 36, 63).

At other inflammatory genes, *T. gondii* alters promoter-associated chromatin although these changes have not been attributed to a specific parasite effector. At the *Tnf* promoter in lipopolysaccharide-stimulated murine bone marrow-derived macrophages, infection reduced recruitment of NF-κB p65, phosphorylated cAMP response element-binding protein (CREB), and phosphorylated c-Jun, blocked H3S10 phosphorylation and H3K9/K14 acetylation, and impaired RNA polymerase II recruitment (64). Nuclear accumulation of NF-κB and CREB was maintained, whereas c-Jun accumulation was reduced, indicating that impaired promoter activation was not attributable solely to defective nuclear translocation (64). At the *Il10* promoter, infection similarly blocked H3S10 phosphorylation and H3K9/K14 acetylation induced by combined Toll-like receptor 4 and Fcγ-receptor signaling (65). Histone crotonylation was altered in a separate porcine alveolar macrophage model, where infection reduced histone H2B lysine 12 crotonylation (H2BK12cr) and increased HDAC2 expression (66). HDAC2 silencing restored both global crotonylation and H2BK12cr (66). For the *Tnf*, *Il10*, and H2BK12cr phenotypes, it is not yet clear whether the chromatin changes are initiated by additional parasite effectors or arise indirectly from infection-induced host responses (64–66).

Host transcriptional states during *T. gondii* infection result from the action of effectors at multiple regulatory levels, including signaling pathways, transcriptional cofactors, corepressor complexes, and chromatin. Genetic evidence now indicates that such activities can operate in combination: TgIST, GRA16, GRA24, and GRA28 jointly protect infected human cells from IFN-γ-driven cell death, while higher-order deletion of eight MYR1-dependent effectors markedly reduces parasite-induced non-canonical NF-κB activation (52, 67). Host transcriptional output is consequently shaped by the collective activity of multiple parasite effectors (12, 52, 67). Individual effector–target interactions capture only part of this response, which varies further across host cell contexts (12, 37, 52, 67). Comparable host chromatin changes have been described in other parasitic infections, although the initiating parasite factors and host regulatory mechanisms differ among systems (see **Box 2**).

Box 2 **Host immune chromatin regulation in parasitic infections beyond *Toxoplasma gondii.***

  Parasite infection and exposure to parasite-derived products alter chromatin states in monocytes and macrophages through distinct regulatory mechanisms. *Leishmania donovani* infection alters host histone methylation at promoters of genes involved in macrophage antimicrobial and regulatory responses. In J774 macrophages and infected BALB/c mice, expression of the methyltransferases ASH1L, SMYD2, and EZH2 and the demethylases KDM5B and KDM6B increased (87). Chromatin immunoprecipitation (ChIP) analysis linked SMYD2-dependent H3K36me2 at the *Tnf* promoter and EZH2-dependent H3K27me3 at the *Nos2* promoter to repression of the corresponding genes (87). KDM5B- and KDM6B-dependent changes in H3K4me3 and H3K27me3 were detected at the *Il12b*, *Tnf*, and *Arg1* promoters, and ASH1L increased H3K4me3 at the *Il10* promoter (87). Perturbation of these histone-modifying enzymes by genetic knockdown or pharmacological inhibition increased pro-inflammatory responses and reduced amastigote multiplication (87).

In *Leishmania amazonensis*-infected macrophages, expression of activating components of the NF-κB–NLRP3 pathway decreased, and expression of negative regulators increased (88). H3K9/K14 acetylation and H3K4me3 were reduced at promoters of NF-κB-regulated pro-inflammatory genes in primary and lesional macrophages (88). The parasite factors that initiate these host chromatin changes are unknown in both *Leishmania* models (87, 88).

The H3K4 methylation response to *Plasmodium falciparum* differs between initial exposure and re-exposure. In malaria-naïve human monocytes, 24 h exposure to infected erythrocytes increased *TNF, IL6*, and *IL1B* expression and enriched H3K4me3 at the *TNF* and *IL6* promoters (89). An independent study detected increased H3K4me1 and H3K4me3 after 4 h exposure to lysed infected erythrocytes, with H3K4me3 enrichment at the same promoters (90). Guha et al. subsequently differentiated exposed monocytes into macrophages and challenged them again with infected erythrocytes. Re-exposed macrophages showed reduced TNF, IL-6, and IL-1β responses together with decreased H3K4me3 at the *TNF* and *IL6* promoters (89).

The glutamate dehydrogenase heGDH from *Heligmosomoides polygyrus bakeri* is internalized by macrophages and contributes to the establishment of chronic infection (91). In human monocyte-derived macrophages, recombinant heGDH increased expression of *IL12B*, *IDO1*, *PTGS2*, *EBI3*, and *PTGES*, together with production of prostaglandin E2, IL-12p40, and IL-10 (91). Inhibition of p300/CBP acetyltransferase activity reduced heGDH-induced gene expression and mediator production and diminished H3K27ac (91). Genome-wide H3K27ac profiling and ChIP followed by quantitative PCR identified heGDH-dependent acetylation changes, including increased H3K27ac at *IDO1*, *PTGS2*, and *IL6* (91).

The mechanistic basis of host chromatin regulation differs among these parasite systems. In *Leishmania*, host histone-modifying enzymes and affected loci have been identified, but the parasite factors that initiate these changes remain unknown (87, 88). *Plasmodium falciparum* exposure alters H3K4me3 at inflammatory loci according to prior exposure history (89, 90). In *H. polygyrus bakeri*, heGDH directly links a defined parasite protein to p300/CBP-dependent H3K27ac and host transcription (91).

## 5 Parasite–host regulatory interactions in immune containment and tissue pathology

The consequences of parasite–host regulatory interactions vary with parasite developmental state, genetic background, and the tissue in which infection occurs (9, 11–14, 38). In the CNS and ocular tissues, loss of cellular immune control permits parasite expansion or reactivation, whereas local inflammatory responses can contribute to neural or retinal injury (6, 7, 68–75). At the maternal–fetal interface, infection alters placental immune regulation and can compromise pregnancy outcomes (76–78). Parasite genotype further modifies host responses through polymorphic effectors such as ROP16 and GRA15, which differ in their effects on host transcription and inflammatory signaling (12–14). The disease relevance of these interactions is particularly evident in two settings: failure of immune containment leading to reactivation (7, 68–70, 79–81), and inflammatory tissue injury that is not necessarily accompanied by a proportional increase in parasite burden (6, 71–75, 82) (**Figure 3**).

**Figure 3:**
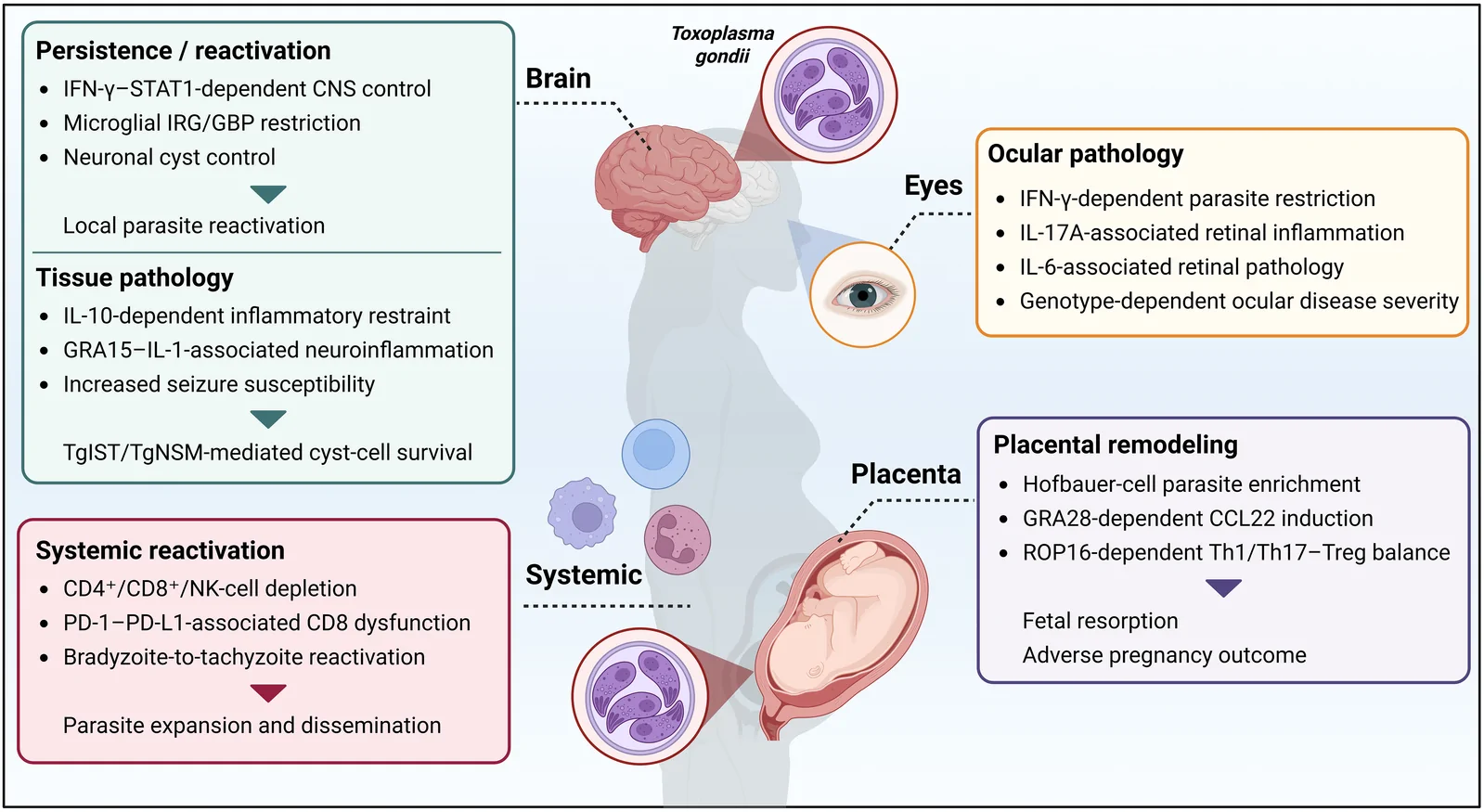
Tissue-specific immune containment and pathology during *Toxoplasma gondii* infection. The schematic summarizes how tissue context and immune status influence the outcomes of *T. gondii* infection in the brain, ocular tissues, systemic compartment, and the placenta. During persistent infection in the brain, IFN-γ–STAT1 signaling supports local parasite control through microglial IRG/GBP responses and neuronal restriction of cysts, whereas loss of immune containment permits local reactivation. CNS pathology is additionally shaped by the regulation of inflammation and parasite effector activity: IL-10 limits encephalitic inflammation, GRA15-dependent IL-1 signaling increases susceptibility to neuroinflammation and seizures, and TgIST and TgNSM suppress necroptotic death of cyst-containing cells. Systemic immune impairment, including depletion or dysfunction of CD4^+^ T cells, CD8^+^ T cells, and natural killer cells, as well as PD-1–PD-L1-associated CD8^+^ T cell dysfunction, favors bradyzoite-to-tachyzoite reactivation, parasite expansion, and dissemination. In ocular toxoplasmosis, IFN-γ contributes to parasite restriction, whereas IL-17A- and IL-6-associated inflammatory responses contribute to retinal pathology; disease severity also varies with parasite genetic background. At the maternal–fetal interface, parasite enrichment in Hofbauer cells is accompanied by placental immune remodeling, including GRA28-dependent CCL22 induction and ROP16-dependent changes in the Th1/Th17–Treg balance, which are associated with fetal resorption and adverse pregnancy outcomes. CNS, central nervous system; GBP, guanylate-binding protein; IFN-γ, interferon-γ; IL, interleukin; IRG, immunity-related GTPase; PD-1, programmed cell death protein 1; PD-L1, programmed death ligand 1; STAT1, signal transducer and activator of transcription 1; Th1, T helper 1; Th17, T helper 17; Treg, regulatory T cell. Image created with BioRender.com.

### 5.1 Loss of immune containment and reactivation of chronic infection

Clinical reactivation of chronic *T. gondii* infection is closely associated with profound impairment of cell-mediated immunity. In HIV-infected individuals, toxoplasmic encephalitis was strongly associated with severe CD4^+^ T cell depletion together with reduced CD8^+^ T cell and natural killer cell numbers, and its incidence declined markedly after the introduction of combination antiretroviral therapy (7). After hematopoietic stem cell transplantation, molecular evidence of reactivation was detected predominantly during the early post-transplant period, before immune reconstitution was complete (68). Recovery of cellular immunity after antiretroviral therapy in patients with previous toxoplasmic encephalitis was accompanied by restoration of *Toxoplasma*-responsive lymphocyte proliferation and antigen-induced IFN-γ production (79). Reactivation during immune loss and recovery of parasite-specific responses after immune reconstitution indicate that control of chronic infection continues to depend on sustained cellular immune competence after tissue cysts have formed (7, 68, 79).

During chronic infection in mice, progressive impairment of CD8^+^ T cell effector and recall responses coincided with increased expression of the tachyzoite-associated marker *ENO2* and reduced expression of the bradyzoite-associated marker *ENO1* (80). Blockade of programmed cell death protein 1 (PD-1)–programmed death ligand 1 (PD-L1) signaling restored CD8^+^ T cell function, shifted parasite expression back toward a bradyzoite-associated profile, reduced parasite dissemination, and prevented mortality in a CD8-dependent manner (80). Deletion of *BFD1* uncoupled cyst formation from long-term parasite persistence. *Δbfd1* parasites failed to establish CNS cysts, yet a low parasite burden was detectable six months after infection (81). Earlier in infection, *Δbfd1*-infected mice had higher levels of tachyzoite replication in the brain, accompanied by severe tissue necrosis and increased mortality compared with mice infected with wild-type parasites (81). Long-term persistence following BFD1 loss was accompanied by increased tachyzoite replication and severe brain necrosis, whereas wild-type-infected brains exhibited inflammation and gliosis without comparable necrotic damage (81).

Bradyzoites export selected effectors, including TgIST, through the MYR1 pathway (11). TgIST impairs STAT1-dependent transcription by interfering with transcriptional coactivator recruitment at IFN-γ-responsive genes (30, 31, 57, 58). Within the CNS, STAT1 signaling in microglia and neurons contributes to parasite control (69, 81). Microglia-specific STAT1 deficiency impaired immunity-related GTPase (IRG)- and guanylate-binding protein (GBP)-mediated antimicrobial responses and permitted marked tachyzoite expansion despite preserved or enhanced T cell IFN-γ responses (69). Neuron-specific *Stat1* deletion increased cyst number and size without a corresponding increase in total parasite burden or tachyzoite replication (81). IFN-γ stimulation enabled murine neurons to eliminate intracellular parasites through IRG-dependent mechanisms and reduced infection in human neurons (70). The consequences of impaired STAT1 signaling differ between CNS cell types. Loss of microglial STAT1 permits tachyzoite expansion, whereas neuronal STAT1 deficiency primarily affects cyst control during chronic infection (69, 81). Whether bradyzoite-exported TgIST alters STAT1-dependent transcription in microglia or neurons has not been tested directly *in vivo*. Control of chronic CNS infection depends on systemic cellular immunity and cell-intrinsic antimicrobial activity in microglia and neurons (7, 68–70, 79–81).

### 5.2 Protective immunity and inflammatory pathology during tissue infection

Loss of cellular immune control permits parasite reactivation or expansion during chronic infection (7, 68–70, 79–81). Tissue pathology differs across ocular, placental, and CNS infection: local cytokine responses shape retinal injury, placental infection alters inflammatory and regulatory programs, and severe CNS inflammation may develop without a parallel increase in parasite burden (71, 72, 74–78). In experimental ocular toxoplasmosis, IL-17A neutralization reduced both intraocular inflammation and parasite multiplication, and concurrent IFN-γ neutralization partially reversed the reduction in parasite growth (71). In a separate ocular reinfection model, IFN-γ neutralization increased parasite burden and aggravated retinal damage, while IL-6 neutralization preserved retinal architecture and reduced the peak parasite burden (72). Across these models, IFN-γ neutralization weakened parasite control (71, 72). In contrast, blockade of IL-17A or IL-6 reduced ocular pathology without increasing parasite burden (71, 72).

Human ocular toxoplasmosis shows substantial heterogeneity in the relationship between local cytokine responses and disease severity. In a comparative study, Colombian patients with more severe disease had higher intraocular parasite loads, lower IFN-γ and IL-17A concentrations, and higher IL-6 and IL-13 concentrations than French patients with milder disease (6). In a separate cohort, primary and recurrent active ocular toxoplasmosis exhibited broadly similar Th1-biased inflammatory profiles (73). Neither lesion size nor vitreous inflammation correlated with individual immune mediators in that cohort (73). Across these studies, no single inflammatory mediator consistently tracked with clinical severity (6, 73).

At the maternal–fetal interface, early human placental explants exposed to *T. gondii* developed inflammatory and chemotaxis-associated transcriptional changes across multiple placental lineages, with most detectable parasite RNA concentrated in fetal Hofbauer cells (76). GRA28 was required for robust CCL22 induction in human and mouse placental explants (77). In a separate murine pregnancy model, deletion of *ROP16* from Chinese 1 WH3 parasites (ROP16I/III/GRA15II) increased placental Th1/Th17-associated responses, reduced regulatory T cell abundance and IL-10/transforming growth factor β1 expression, and increased fetal resorption (78). The contribution of ROP16-dependent placental immune changes to fetal resorption warrants direct testing (78).

During chronic CNS infection, IL-10 restrains inflammatory pathology without a corresponding reduction in parasite burden (74, 75). IL-10-deficient mice developed lethal encephalitic inflammation despite parasite burdens comparable to those of wild-type mice, and partial depletion of CD4^+^ T cells markedly attenuated pathology and restored survival (74). IL-10 receptor blockade after establishment of chronic infection similarly provoked severe CNS inflammation, accompanied by expansion of CD4^+^ effector T cells and increases in antigen-presenting cell activation, inflammatory cytokine production, and neutrophil recruitment. Brain cyst numbers remained unchanged (75). By contrast, inducible T cell costimulator ligand blockade expanded brain T cell populations without producing comparable tissue destruction (75). Within infected cells, combined loss of TgNSM and TgIST increased IFN-γ-induced host cell death and rupture of intracellular cysts (33). During chronic type II infection, GRA15-dependent IL-1 signaling in the brain was associated with increased susceptibility to chemically induced seizures (82). Loss of GRA15 reduced both the inflammatory transcriptional response and seizure severity, while pharmacological blockade of IL-1 receptor signaling attenuated seizures without altering chronic cyst burden (82). Across these tissues, parasite restriction, inflammatory injury, and effector-mediated host regulation jointly influence disease outcome (6, 33, 71–78, 82).

## 6 Concluding perspectives

The transcriptional and chromatin control of *T. gondii* development and the mechanisms of individual host-modulatory effectors have been defined in considerable detail, but direct regulatory links between developmental state and effector output remain sparse (3, 9–11, 20, 21, 49, 52). Chromatin remodelers and ApiAP2 factors establish stage-appropriate transcriptional states, while developmental transitions are accompanied by selective changes in host-modulatory effector expression and delivery (3, 9–11, 15–22, 26). For most effectors, however, direct control by developmental transcriptional or chromatin regulators has not been established, and effector abundance does not necessarily predict delivery into the host cell (10, 11, 20, 26). Once delivered, effectors act at multiple levels of host gene regulation, including signaling pathways, STAT-dependent transcription, transcriptional cofactor and corepressor systems, and chromatin regulation (13, 14, 30, 31, 33–36, 52–55, 57). Higher-order effector perturbations indicate that host transcriptional output reflects the combined activity of multiple parasite effectors (52, 67). The consequences of host–parasite regulation differ across tissues. Loss of cellular immune control permits parasite expansion or reactivation (7, 68–70, 79–81), while inflammatory pathology in ocular, placental, and CNS infection is influenced by local immune responses and parasite effector activity (6, 71–78, 82).

Directly connecting parasite developmental regulation to effector activity requires experiments that distinguish direct regulation of effector genes from secondary transcriptional changes accompanying stage conversion. Acute perturbation of candidate regulators combined with mapping of regulator binding sites and measurement of transcriptional responses would help distinguish directly regulated effector genes from indirect targets. Effector abundance and host-cell delivery require separate assessment because their kinetics differ during differentiation (9–11). Perturbation-coupled host–parasite profiling could link defined effector changes to host transcriptional responses while accounting for host cell identity (37, 83). Parallel measurements of chromatin accessibility or histone state would then test whether these transcriptional changes are accompanied by corresponding changes in host chromatin. Such analyses should ultimately extend across parasite developmental states, genetic backgrounds, host cell types, and infected tissues to determine which regulatory relationships operate *in vivo* (9, 12, 14, 37). The central challenge is to establish direct links from parasite regulatory state to effector repertoire and from effector repertoire to tissue-specific host transcriptional and chromatin responses that influence immune containment and pathology.
